# Association of Anthropometry With Nerve Conduction Parameters of Median Nerve: A Cross-Sectional Study in a North Indian Medical University Hospital

**DOI:** 10.7759/cureus.63946

**Published:** 2024-07-06

**Authors:** Rachna Singh, Shubhajeet Roy, Archna Ghildiyal, Shivam Verma

**Affiliations:** 1 Medicine, Faculty of Medical Sciences, King George's Medical University, Lucknow, IND; 2 Surgery, Faculty of Medical Sciences, King George's Medical University, Lucknow, IND; 3 Physiology, King George's Medical University, Lucknow, IND

**Keywords:** sensory, motor, amplitude, velocity, latency, median nerve, nerve conduction study

## Abstract

Background

Nerve conduction studies ease the understanding of the various pathologies of the peripheral nervous system. It helps physicians to delineate between the two principal types of peripheral etiologies: axonal degeneration and demyelination. An increase in weight in the form of excessive fat deposition or obesity could have a worrisome effect on nerve conduction. So, to find the association of various anthropometric parameters (age, gender, height, weight, waist-hip ratio and body mass index) with motor and sensory median nerve conduction parameters (latency, amplitude and velocity) this cross-sectional study was conducted.

Materials and method

A total of 87 subjects were taken and their height, weight, waist-hip ratio and body mass index were measured using standard techniques. Motor and sensory nerve conduction parameters were measured on an electromyography machine. Data was stored, tabulated and analyzed.

Results

The average height of male and female subjects ± SD was 1.699 ± 0.072 m and 1.589 ± 0.067 m respectively. The average weight of male and female subjects ± SD was 64.089 ± 11.497 kg and 52.949 ± 8.404 kg, respectively. The average BMI of normal, underweight and overweight subjects ± SD was 21.668 ± 2.048 kg/m^2^, 17.074 ± 0.794 kg/m^2^ and 26.595 ± 0.915 kg/m^2^ respectively. Weight showed a significant (p = 0.0025) correlation with the latency of motor median nerve conduction. Waist-hip ratio showed a significant (p = 0.042 and p = 0.036) correlation with motor median nerve conduction velocity in both male and female subjects, respectively. BMI in the overweight category showed a significant (p = 0.0156 and p = 0.0290) correlation with latency and amplitude of motor median nerve conduction study, respectively.

Conclusions

This study exemplifies that an increase in BMI of our body can affect nerve conduction. This could serve as a preliminary study to assess the effect of obesity on peripheral nerve conduction, especially in the Indian population.

## Introduction

In the 21st century obesity has emerged as an epidemic, which has doubled since 1980, especially in developing countries like India, where it is continuously rising and occurring more among youngsters [1,2]. The major causes of obesity are an increase in intake of energy-dense food, that is high in fat, decreased physical activity, and increasing urbanization with changes in lifestyle.

Nerve conduction velocity (NCV) is a test by which we estimate the nervous velocity and electrical activity, and it has been used to observe nerve function, ascertain the progression of disease, evaluate the complications in the treatment, as well as to identify the disease [3].

Nerve conduction studies (NCS) help in assessing peripheral motor and sensory functions by recording the response to electrical stimulation of peripheral nerves. Nerve conduction differs in various age groups [4,5] and a negative association with increasing age was reported. As far as other factors are concerned, varying observations have been made in NCS between males and females [6,7]. Research focusing on the relation between height and NCV also differs amongst themselves, and results are therefore quite controversial [7].

Much of the existing literature takes into account the elements that influence nerve conduction velocities, which are classified into physical and biological factors (age, height and gender) [8-10]. Soudmand et al. (1993) observed that there was no relationship between both motor and sensory components of the median nerve’s conduction velocity and height [11]. On the other hand, Naseem et al. (2016) observed a relatively significant association between NCV and height in both males and females [12]. Awang et al. (2006) found that there was no significant relation in median sensory conduction velocity in different age groups, and also observed a reduction in median motor conduction velocity with increasing age, which was significant [13]. Saeed and Akram (2008) described in their study an insignificant gender-related difference in the conduction studies of the sural nerve [5]. Whereas Robinson et al. (1993) concluded that females had significantly faster conduction velocities than men for all nerves except the median motor nerve in their study [14]. Buschbacher (1988) in his study of the effect of body mass index on NCV found that there was no significant correlation between BMI and NCV of the median nerve [15]. In a study by Chadha and Shivalkar (2016), sensory median nerve amplitudes were found to be decreasing with increasing BMI, but there was no significant correlation between BMI and NCV, or latency [16]. In a study done on the Indian population, weight and BMI did not show a significant association with nerve conduction parameters as seen by Pawar et al. (2012) [10].

The majority of the studies have been performed on Western populations and varying results exist in the literature, hence this study was planned, which has tried to establish a relation between various anthropometric parameters (height, weight, body mass index, waist-hip ratio) to NCV and other parameters, like latency and amplitude of both the motor and sensory components of the median nerve, in an Indian population and draw out necessary conclusions.

## Materials and methods

This cross-sectional study was conducted at the Department of Physiology in a Tertiary Care Medical University of Northern India. This study included subjects of the age group 18 to 25 years, irrespective of sex, without any addiction to tobacco or alcohol. Before including, the consent of the subject was obtained. The exclusion criteria included subjects of age above 25 years or below 18 years, subjects with a history of chronic diseases like diabetes, hypertension, neurological disorders, and subjects taking medications.

The sample size was determined using the Cochran’s sample size formula, considering 95% confidence level, 5.5% error, and 7.26% population proportion. According to this calculation, the sample size comes out to be 86. A convenient sampling method was used.

A total of 87 subjects were taken and their anthropometric parameters were measured using standard techniques after obtaining their consent. An electromyography machine was used to measure the nerve conduction parameters on their dominant hand. For the motor nerve conduction study, two electrodes were used. The reference electrode was kept on the tendon of the abductor pollicis brevis muscle, and the active electrode was placed on the belly of the abductor pollicis brevis muscle. Two stimuli of 30 mA were given, one on the dominant wrist and the other in the cubital fossa on the skin of the elbow. To calculate the distance between the wrist and elbow, measuring tape was used. To obtain conduction velocity, the difference in the latencies between the responses evoked by nerve stimulation at the wrist and elbow was calculated. Latency and amplitude were also recorded. For the sensory nerve conduction study, the same reference and active electrodes were placed on the distal interphalangeal joint and metacarpophalangeal joint of the index finger, respectively. Stimulations of 10 mA were given. Conduction velocity was measured. Latency and amplitude were also calculated and presented in a tabulated form.

The data was saved in Microsoft Excel 2019. SPSS (Statistical Package for Social Science) version 25 (IBM Corp., Armonk, NY, USA) was used to perform the higher statistical analysis. t-test for two independent means was used to assess the difference between various parameters (latency, amplitude and velocity) of both motor and sensory median nerve conduction study among males and females. We used Karl Pearson's correlation to study the correlation between various anthropometric parameters (age, gender, height, weight, waist-hip ratio and body mass index) and NCV and other parameters (latency and amplitude) of motor and sensory median nerve conduction study (NCS). A p-value of less than 0.05 was considered statistically significant.

The study was conducted after approval by the Institutional Ethics Committee (Reference code: XI-PGTSC-I B/P1, dated: 2022 Aug 04).

## Results

The cross-sectional study was conducted on 87 (n). The study included 48 boys and 39 girls enrolled in 1st year of MBBS course at a Tertiary Care Medical University of Northern India. The average age of the individuals was 20.402 ± 1.622 years.

The comparative motor and sensory median nerve conduction study in boys and girls has been shown in Table 1. For comparison between boys and girls, the t-test for two independent means was used.

**Table 1:** The comparison of motor and sensory median nerve conduction study in boys and girls Where Lm, Am and Vm are the Latency, Amplitude and Velocity of the motor component of the median nerve conduction study, respectively, and Ls, As and Vs are the Latency, Amplitude and Velocity of the sensory component of the median nerve conduction study, respectively. * indicates p-values (p<0.05) that are statistically significant.

Mean ± SD	Girls	Boys	t-value	p-value
Lm (mS)	2.802 ± 0.256	3.042 ± 0.351	-3.456	.00086*
Am (mV)	14.070 ± 3.507	13.124 ± 3.723	2.792	.00648*
Vm (m/s)	58.675 ± 4.810	56.165 ± 4.004	.901	.1850
Ls (mS)	2.139 ± 0.279	2.232 ± 0.205	-1.62	.107
As (μV)	83.043 ± 32.400	72.357 ± 61.670	1.96	.052
Vs (m/s)	61.605 ± 6.262	58.782 ± 6.128	1.033	.30

Based on their BMI, the subjects were categorized into Underweight (UW) (n1=8), Normal (N) (n2=68) and Overweight (OW) (n3=11). The average BMI of normal subjects was 21.668 ± 2.048 kg/m^2^, that of underweight subjects was 17.074 ± 0.794 kg/m^2^ and for overweight individuals it was 26.595 ± 0.915 kg/m^2^.

The influence of BMI on nerve conduction velocity and other parameters (Latency and Amplitude) of motor and sensory median nerve conduction study has been shown in Table 2. Only latency and amplitude of the motor component in overweight individuals were found to have a significant correlation with the BMI.

**Table 2:** The influence of BMI on nerve conduction study (NCS) parameters Where Lm, Am and Vm are the Latency, Amplitude and Velocity of the motor component of the median nerve conduction study, respectively, and Ls, As and Vs are the Latency, Amplitude and Velocity of the sensory component of the median nerve conduction study, respectively. * indicates p-values (p<0.05) that are statistically significant.

NCS Parameter	UW	r-value	p-value	N	r-value	p-value	OW	r-value	p-value
Lm (mS)	3.056±0.192	.456	.447	2.879±0.332	.178	.150	3.187±0.282	.703	.0156*
Am (mV)	13.32±4.041	-.040	.925	13.914±3.483	-.044	.725	11.452±3.704	-.654	.0290*
Vm (m/s)	56.44±3.786	-.263	.529	57.742±4.515	.241	.051	55.112±4.616	.579	.0619
Ls (mS)	2.225±0.103	-.031	.940	2.157±0.256	-.059	.637	2.373±0.153	.218	.0518
As (μV)	89.275±25.93	-.575	.135	78.514±56.172	-.136	.276	59.876±11.391	.064	.456
Vs (m/s)	58.551±2.736	-.210	.617	61.042±6.584	.026	.835	54.987±3.425	.535	.320

The average BMI of boys was 22.632±2.894 kg/m^2^ and that of girls was 20.979±2.678 kg/m^2^. The correlation between BMI and NCS parameters in boys and girls has been shown in Table 3.

**Table 3:** The correlation between BMI and nerve conduction study (NCS) parameters Where Lm, Am and Vm are the Latency, Amplitude and Velocity of the motor component of the median nerve conduction study, respectively, and Ls, As and Vs are the Latency, Amplitude and Velocity of the sensory component of the median nerve conduction study, respectively. * indicates p-values (p<0.05) that are statistically significant.

NCS Parameter	Overall		Boys		Girls	
	r-value	p-value	r-value	p-value	r-value	p-value
Lm (mS)	.2338	.0318	-.0393	.792	.3344	.043*
Am (mV)	-.1677	.1266	-.1834	.213	-.2117	.2099
Vm (m/s)	.0416	.705	.028	.850	-.092	.588
Ls (mS)	.1094	.320	.4019	.004*	.1673	.322
As (μV)	-.1954	0.073	-.0424	.702	-.1748	.303
Vs (m/s)	-.1252	0.254	-0.1996	0.067	-0.1427	0.401

The average overall height was 1.660±0.088 m. The average height of boys was 1.699±0.072 m, and that of girls was 1.589±0.067 m. The correlation between height and NCS parameters has been shown in Table 4.

**Table 4:** The correlation between height and nerve conduction study (NCS) parameters Where Lm, Am and Vm are the Latency, Amplitude and Velocity of the motor component of the median nerve conduction study, respectively, and Ls, As and Vs are the Latency, Amplitude and Velocity of the sensory component of the median nerve conduction study, respectively. * indicates p-values (p<0.05) that are statistically significant.

NCS parameters	Overall		Boys		Girls	
	r-value	p-value	r-value	p-value	r-value	p-Value
Lm (mS)	.4196	.064	.2566	.0752	.2637	.1104
Am (mV)	.0054	.960	.2419	.0923	-.0743	.0622
Vm (m/s)	-.2792	.771	-.0705	.2346	-.1405	.2750
Ls (mS)	.1518	.1677	.1861	.2004	-.0881	.6018
As (μV)	-.1477	.179	-.0988	.5462	-.1093	.9200
Vs (m/s)	-.2022	.063	-.6742	.3466	-.0281	.6654

The overall average weight was 60.718±11.834 kg. The average weight of boys was 64.089±11.497 kg, and that of girls was 52.949±8.404 kg. The correlation between weight and NCS parameters has been shown in Table 5.

**Table 5:** The correlation between weight and nerve conduction study (NCS) parameters Where Lm, Am and Vm are the Latency, Amplitude and Velocity of the motor component of the median nerve conduction study, respectively, and Ls, As and Vs are the Latency, Amplitude and Velocity of the sensory component of the median nerve conduction study, respectively. * indicates p-values (p<0.05) that are statistically significant.

NCS Parameter	Overall		Boys		Girls	
	r-value	p-value	r-value	p-value	r-value	p-value
Lm (mS)	.0828	.455	-.0089	.0025*	.4251	.062
Am (mV)	-.158	.1486	-.087	.635	-.0849	.573
Vm (m/s)	.1601	.1435	.2489	.137	-.1016	.492
Ls (mS)	.0586	.594	-.0617	.714	.2339	.104
As (μV)	-.0295	.7921	-.2111	.209	-.2018	.170
Vs (m/s)	-.0751	.495	.0198	.907	-.2043	.164

Waist:Hip Ratio (WHR) was classified according to the study by Bray et al. into Good, Average and At risk population [17]. Average WHR in boys and girls were 0.842±0.543 and 0.786±0.062, respectively. The correlation between WHR and NCS parameters has been shown in Table 6.

**Table 6:** The correlation between waist:hip ratio (WHR) and nerve conduction study (NCS) parameters Where Lm, Am and Vm are the Latency, Amplitude and Velocity of motor median nerve conduction study, respectively. Ls, As and Vs are the Latency, Amplitude and Velocity of sensory median nerve conduction study, respectively. * indicates p-values (p<0.05) that are statistically significant. None of the male subjects had a waist-hip ratio greater than 0.95 hence the third category was omitted.

NCS Parameter	Overall		Girls						Boys			
			Good WHR (0.75-0.79)		Average WHR (0.8-0.86)		At risk WHR (>0.86)		Good WHR (0.85-0.89)		Average WHR (0.9-0.95)	
	r-value	p-value	r-value	p-value	r-value	p-value	r-value	p-value	r-value	p-value	r-value	p-value
Lm (mS)	-.0847	.444	-.5684	.1180	.441	.131	.1672	.788	-.1113	.494	-.3090	.456
Am (mV)	-.066	.367	.1517	.535	-.5519	.0509	.455	.441	-.1709	.294	-.1752	.678
Vm (m/s)	.099	.367	.3848	.103	-.5839	.036*	-.5132	.376	.3227	.042*	-.0199	.964
Ls (mS)	.1238	.262	-.1518	.537	.4620	.119	.1396	.822	.2484	.122	-.1442	.733
As (μV)	-.005	.9637	.0975	.691	-.0338	.914	.4428	.455	.1212	.456	.4783	.230
Vs (m/s)	-.181	.0973	.181	.458	.458	.009	-0.1601	0.797	-.2523	.116	.1346	.750

## Discussion

In our study, we included age, gender, height, weight, waist-hip ratio and BMI and studied their influence on various parameters of both motor and sensory median nerve conduction study (latency, amplitude and velocity) on the dominant hand. Owing to temperature or diurnal rhythm, the study was conducted during a fixed daytime to prevent any kind of bias. All the participants were of similar age group, to rule out any kind of age-related bias [18], as was observed in a study by Tong et al., where it was seen that median sensory velocities decreased at a rate of 0.14 m/year of age [19]. Werner et al. also concluded a decrease in conduction velocity with increasing age in their study [20].

In our study, we observed a non-significant correlation between height and NCS parameters in both male and female subjects. In contrast to our findings, Hennessey et al. showed a negative correlation of height with median nerve sensory amplitude and conduction velocities, and a positive correlation with latency in his study [21]. Also, Naseem et al. found a significant association of height and NCV in both male and female subjects [12].

A further study done on the correlation of weight and NCS parameters (between boys and girls) showed a significant positive correlation between latency of motor median nerve conduction study in boys, hypothesizing that as the body weight increases due to an increase in subcutaneous fat deposition around the limbs, the latency increases as well. However, in the study done by Pawar et al. on the Indian population, they showed that weight and BMI have an insignificant influence on nerve conduction parameters [22]. In our study, a significant correlation was found only with motor component latency in boys (p=0.0025).

In our study, it was found that girls had a greater mean conduction velocity compared to males. A statistically significant difference was seen in the values of median motor nerve conduction velocity and its latency among male and female subjects. In contrast to our findings, Soudmand et al. observed in their study that NCV and distal latency weren’t influenced by gender [11]. The difference in the NCV findings with respect to gender can be probably explained by the difference in body fat percentage between males and females, as body fat content has an influence on the nerve conduction. However, the previous study by Soudmand et al. didn't note any such finding, because gender is not the only factor dictating body fat percentage, and also the body fat percentage is not the only variable on which NCV is dependent upon. The results of various studies depend a lot on the subject selection and sample characteristics, which might vary from study to study, region to region, and race to race, thereby influencing the results of such studies.

In this current study, the waist-hip ratio was seen to have a negative significant correlation between motor nerve conduction velocity in both boys and girls in good and average categories, respectively. This observation suggested that as the waist-hip ratio increases there will be a decrease in the velocity due to greater fat deposition around the waist, although there is no previous literature to support this hypothesis.

Awang et al. had shown that there was a reduction in sensory NCV with an increase in BMI [13]. In our study, we observed that there was a significant positive correlation between BMI and latency of median motor nerve conduction in overweight subjects. The above finding is supported by a similar observation in weight (positive correlation with the latency of motor median nerve conduction study in males). This hints towards the fact that as the BMI increases due to an increase in weight it will lead to an increase in the latency of motor median nerve conduction. Furthermore, a significant negative correlation was observed between BMI and amplitude of motor median nerve conduction study in overweight subjects. This could be due to greater subcutaneous fat deposition around the limbs. No significant correlation was observed between BMI and motor median nerve conduction velocity and sensory median nerve conduction parameters. Pawar et al. in their study had observed that weight and BMI have an insignificant effect on nerve conduction parameters in the Indian population [22].

Limitations

First of all, the study included only 87 subjects, which may limit the generalizability of the results. A greater sample size would have provided a more robust and representative data. Secondly, it being a cross-sectional study, it could only establish associations rather than any causation. Cohorts will be perfect studies to determine causal relationships between anthropometric parameters and NCV. Thirdly, the study concentrates on a North Indian population, which can limit the applicability of the results to other ethnic groups and populations in other regions with different genetic, racial and environmental backgrounds. Fourthly, our study does not include other variables that might have an effect on nerve conduction, like physical activity, diet, or metabolic disorders. Lastly, the accuracy of the nerve conduction measurements may have been influenced by technical variability or operator-dependent factors. Standardization and calibration of the electromyography machine can vary from laboratory to laboratory.

## Conclusions

From the above study, we conclude that there was no significant correlation between height and motor and sensory median nerve conduction parameters. Weight showed a positive significant correlation with the latency of motor median nerve conduction study in males. There was a significant difference in the values of median motor nerve conduction velocity and its latency among male and female subjects. Female subjects with a good waist-hip ratio had a negative significant correlation with motor nerve conduction velocity while male subjects with an average waist-hip ratio also showed a negative significant correlation with motor nerve conduction velocity. BMI of overweight subjects showed a positive significant correlation with the latency of the motor median nerve conduction study and a negative significant correlation with the amplitude of the motor median nerve conduction study. This study adds further evidence to the literature to support the hypothesis that an increase in fat deposition in our body can affect nerve conduction. This study could be used as a preliminary study to assess the effect of obesity on the peripheral nerve conduction in the Indian population.
